# Evaluation of Micro-Mechanism and High- and Low-Temperature Rheological Properties of Disintegrated High Volume Crumb Rubber Asphalt (DHVRA)

**DOI:** 10.3390/ma14051145

**Published:** 2021-02-28

**Authors:** Wei Li, Sen Han, Xiaokang Fu, Ke Huang

**Affiliations:** 1 Highway and Airport Pavement Research Center, School of Highway, Chang’an University, Xi’an 710064, China; lhdww_forever@chd.edu.cn; 2 Shanxi Jingxing Expressway Co., Ltd., Luliang 033000, China; XKF253@163.com; 3 Road and Bridge Design and Research Institute Branch of China Highway Engineering Consulting Group Co., Ltd., Wuhan 430024, China; kh1552@126.com

**Keywords:** modified asphalt, high volume crumb rubber, disintegrating agent, micro-mechanism, rheological characteristics

## Abstract

The aims of this paper are to prepare disintegrated high volume crumb rubber asphalt (DHVRA) with low viscosity, good workability and low-temperature performance by adding disintegrating agent (DA) in the preparation process, and to further analyze the disintegrating mechanism and evaluated high-temperature and low-temperature rheological properties. To obtain DHVRA with excellent comprehensive performance, the optimum DA dosage was determined. Based on long-term disintegrating tests and the Fluorescence Microscopy (FM) method, the correlations between key indexes and crumb rubber (CR) particle diameter was analyzed, and the evaluation indicator and disintegrating stage division standard were put forward. Furthermore, Fourier transform infrared spectroscopy (FT-IR) and Gel Permeation Chromatography (GPC) was used to reveal the reaction mechanism, and the contact angle test method was adopted to evaluate the surface free energy (SFE). In addition, the high-temperature and low-temperature rheological properties were measured, and the optimum CR content was proposed. Results indicated that the optimum DA dosage was 7.5‰, and the addition of DA promoted the melt decomposition of CR, reduced the viscosity and improved the storage stability. The 135 °C rotational viscosity (RV) of DHVRA from mixing for 3 h could be reduced to 1.475 Pa·s, and the softening point difference was even less than 2 °C. The linear correlation between 135 °C RV and the diameter of CR particle in rubber asphalt system was as high as 0.968, and the viscosity decay rate (VDR) was used as the standard to divide the disintegrating process into a fast disintegrating stage, stable disintegrating stage and slight disintegrating stage. Compared to common rubber asphalt (CRA), DHVRA has an absorption peak at 960 cm^−1^ caused by trans olefin = C-H, and higher molecular weight and polar component of surface energy. Compared with CRA, although the high-temperature performance of DHVRA decreases slightly, the low-temperature relaxation ability can be greatly improved.

## 1. Introduction

Waste tires have strong heat resistance, mechanical resistance and degradation resistance. If they are piled up for a long time, it will not only cause a waste of land resources but can also cause fire, breed mosquitoes and spread diseases [1]. The way to use waste tires effectively has become an urgent problem. According to a report, the United States, Europe and Japan and other developed countries mainly use waste tires to burn heat and produce crumb rubber (CR) [2]. Moreover, the annual output of waste tires in China is about 12 million tons, and 70% of waste truck tires were used to produce recycled rubber and modified asphalt [3,4]. With the rapid growth of the economy and the promotion of sustainable development policy, rubber asphalt (RA) has gradually developed and has been applied all over the country [5,6,7,8]. For common rubber asphalt (CRA), it is difficult to get the content of CR to exceed 25% [9,10]. 

Despite existing research showing that CRA could improve the rutting resistance and noise reduction and prolong the service life of pavement [11,12,13,14], there is still a series of problems, such as easy segregation, high viscosity and poor performance at low-temperature [15,16]. In this context, some measures should be taken to improve its performance deficiencies. Existing research has indicated that CR can be pretreated by various methods to effectively improve the compatibility with the base binder and obtain RA with high CR volume [17,18,19]. Considering the maximum utilization of waste CR, high volume crumb rubber asphalt (HVRA) is prepared by pretreatment of CR, and the CR content is more than 20%. The ordinary CR pretreatment methods for HVRA can be summarized including thermal-mechanical methods and chemical methods [20,21,22,23,24,25]. Zhang et al. prepared waste CR by a twin-screw extrusion method, and the CR content was as high as 20–40% [21]. The chemical method was combined with a mechanical method due to the variety of additives and complicated principle involved [23]. Wang et al. [24,25] used sodium hypochlorite, activator, gasoline, and diesel oil to pretreat CR, and the CR content was up to 30–49%. Although the use of additives significantly improved the compatibility of base binder and CR and greatly increased the amount of CR, the modified asphalt still had a high viscosity and poor storage stability at high temperature. Additionally, the commonly used a modified asphalt improvement process that included changing the heating temperature, mixing time, shear rate and other preparation processes. Zheng [25] increased the heating temperature to 270 °C and extended the mixing time to evaluate the influence of high-temperature pyrolysis process on the storage stability of HVRA. Although a preparation method of HVRA has been proposed, it needed to be carried out under the joint action of desulfurizer, softener, vulcanizing agent, and vulcanizing softener, and the preparation process is complex [26].

Until now, the research on HVRA has focused on how to improve the CR content and the preparation technology by various means. Most of the HVRA, like CRA, still has some problems such as poor storage at high temperature and high viscosity [27]. Additionally, CR has its own complex pyrolysis characteristics, including irregular chain breaking, chain stripping, cross-linking of bonds and coking reaction [28]. Glover et al. studied the degradation and anti-vulcanization of CR in asphalt and found that 90% CR was dissolved in asphalt with high-speed shear for 40 min and treatment for 6.5 h at 260 °C [29]. Liu et al. concluded that when the pyrolysis temperature of the CR was higher than 200 °C, the CR began to decompose [30]. However, high-temperature reaction and long-time pyrolysis reaction, on the one hand, increased the energy consumption and prolonged the preparation period and, on the other hand, increased the aging of asphalt phase and had a negative effect on the performance of asphalt. In this case, stable RA was prepared by activated CR, and the disintegrating-polymerization process was adopted to improve the comprehensive properties of RA by adding disintegrating agent (DA) in the rubber granulation process and adding polymer modifier in the preparation process [31]. 

Rubber as a kind of polymer network structure material is an elastomer with a huge cohesive force. Therefore, it is difficult to disperse a large amount of CR evenly in base binder by simple thermal and mechanical energy [32,33]. The conventional production method is to desulfurize the CR in the special desulfurizing equipment first and then add the desulfurized CR into VA [33]. In this way, the preparation process of RA is complex, which not only has high cost but also has low efficiency and increases the probability of environmental pollution. Therefore, considering the properties of HVRA and disintegrated RA comprehensively, this paper puts forward the disintegrated high-volume crumb rubber modified asphalt (DHVRA). Compared to CRA, DHVRA has the following obvious advantages: (1) The high CR content (>20% by the weight of base binder) makes full use of waste CR resources and brings higher environmental and economic benefits. (2) The viscosity of asphalt is essential to its workability for applications in civil engineering [34]. Under the action of DA, rubber particles can be rapidly disintegrated into smaller particles in a shorter time and be more evenly dispersed in the base binder phase. It not only improves the storage stability of RA system but also reduces the high-temperature viscosity to a certain extent, further improving the workability. (3) Asphalt pavement paved with high-penetration asphalt binder has lower stiffness modulus and less cracking than that with low-penetration asphalt. The penetration of DHVRA is higher, and the low-temperature performance can be improved significantly. 

In this study, combined with an explanation of the advantages of DHVRA, a homogeneous DHVRA with good storage stability, low viscosity and good low-temperature relaxation ability is obtained. The main outcomes were as follows: (1) Through long-term disintegrating tests, the optimum indexes to evaluate the performance of DHVRA were determined. (2) The micro-disintegrating mechanism of DHVRA was analyzed by fluorescence microscope (FM), Fourier transform infrared spectroscopy (FT-IR), Gel chromatography (GPC) and contact angle test. (3) The high-temperature and low-temperature rheological properties of DHVRA were studied to provide a theoretical basis for widely recommending DHVRA to field engineering.

## 2. Materials and Methods 

### 2.1. Materials

#### 2.1.1. Virgin Asphalt (VA)

The different content of light components in virgin asphalt (VA) will influence the disintegrating effect of CR in RA. Existing studies have concluded that VAs with a high content of light components are conducive to the dissolution of crumb rubber [35]. Moreover, the influence of aromatics on the disintegrating degree of CR in RA was greater than that of other components. This paper focused on the study of disintegrating law of DHVRA, a VA with a high content of light components, and showed that especially aromatic components should be chosen. According to the Chinese standard JTG E20-2011 [36], the analysis of four components of different asphalt was determined, and the SK-90 was finally selected. Four components of different VAs and main indicators of SK-90 are shown in Table 1 and Table 2, respectively.

#### 2.1.2. Crumb Rubber (CR)

Different sizes of CR will differently affect the performance of RA. When the size was more than 40 mesh, it was more favorable for desulfurization and degradation, and the performance of RA at high temperature and low temperature was also better. This paper studied the RA with high content CR; the smaller the size of CR, the higher the cost. In this case, a CR with 40 mesh produced by Changzhou Rongao chemical new material Co., Ltd. was selected, and three contents (24%, 28%, and 32% by the total weight of base binder) were adopted. The main indicators of 40 mesh CR are shown in Table 3.

#### 2.1.3. Disintegrating Agent (DA)

Due to the vulcanization treatment of the original rubber material in the process of tire production, a large number of double bond structures of the rubber material are opened. Therefore, the surface of the CR material produced by waste tires is inert, and it is difficult to mix the rubber particles with base binder evenly through simple mechanical mixing. In order to change this disadvantageous state, adding an appropriate amount of DA in the process of RA preparation can increase the surface energy and chemical energy of CR to a certain extent. As a result, it can enhance its polarity and reactivity, promote the full reaction between the base binder and CR, and make CR particles degrade into small particles and mix with asphalt evenly at high temperature. 

**Figure 1:**
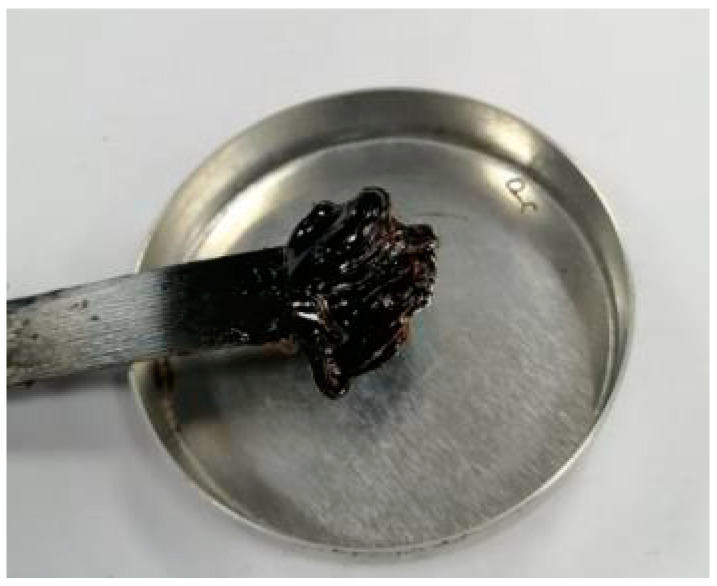
Disintegrating agent at ambient temperature.

In this paper, the ammonium phosphate DA was used. It is a dark brown solid at ambient temperature (as shown in Figure 1), and the density is 1.038 g/cm^3^. The five DA contents (7.5‰, 5‰, 7.5‰, 10‰, 15‰ and 20‰ by total weight of VA) were selected.

### 2.2. Preparation of DHVRA

FM 300 high-speed shear machine was used to prepare RA. Before preparation, preheat the weighed CR in the oven at 105 °C for 15 min and heat the VA at the same time. When the temperature of the base asphalt was about 160 °C, the CR and DA were added into VA simultaneously, and the shear speed was about 800 rpm. After mixing for 20 min, the mixing temperature rose to 190 °C and remained stable. Mixing continued for 1 h, and then the shear rate increased to 1800 rpm and stirring continued for 0.5 h, 1 h, 1.5 h, 2 h, 2.5 h, 3 h, 4 h, 5 h, 8 h, 12 h and 18 h, respectively, to study the influence of mixing time on asphalt performance.

### 2.3. Test Methods

#### 2.3.1. Analysis of Technical Characteristics of DHVRA

##### Analysis of Long-Term Disintegrating Tests of DHVRA

To fully reveal the disintegrating reaction process and determine the optimal reaction time, a long-term disintegrating test was conducted. The change of penetration and rotational viscosity (RV) with the extension of mixing time could indirectly reflect the degree of disintegrating reaction and the change in disintegrating products. The change rate of penetration and RV could be presented by penetration growth rate (PGR) and viscosity decay rate (VDR). The larger the PGR and VDR, the faster the penetration increased and the more the RV decreased. PGR and VDR were calculated as Equations (1) and (2). The parameters of long-term disintegrating tests are shown in Table 4.

(1) $$\mathrm{PDR}=(P_{t2}-P_{t1})\times 100/P_{t1}$$

(2) $$\mathrm{VDR}=(V_{t2}-V_{t1})\times 100/V_{t1}$$

where $P_{t1}$, $P_{t2}$ are penetrations of previous and subsequent time period, respectively. $V_{t1}$, $V_{t2}$ are RV of previous and subsequent time period, respectively.

##### Storage Stability

Softening point difference was used to evaluate the storage stability of DHVRA. The separation test was carried out according to the Chinese requirements of JTG E-20-2011 [36]. A 25 mm diameter and 140 mm long aluminum tube with one end open was used to weigh about 50 g of DHVRA, and then it was put into an oven at 163 °C for 2 h, 4 h and 6 h respectively. After cooling, 1/3 of the samples at the top and bottom of the aluminum tube were cut off respectively, and the asphalt inside the cut aluminum pipe was taken out. Then, the softening point was tested, and the softening point difference was calculated. Finally, the storage stability of DHVRA and CRA was compared and analyzed.

##### Fluorescence Microscope (FM)

BK-FL fluorescence microscope (FM) and the associated Toup view software and imaging device were used for image acquisition. The magnification of the observed object was 200 times. The FM images of DHVRA after mixing for 1 h, 3 h, 5 h and 18 h were acquired. The Image Pro Plus (IPP) software was used to analyze and quantify the FM images. IPP can mark the area of interest (AOI) in the image and determine the max diameter, min diameter and mean diameter of the target area.

**Figure 2:**
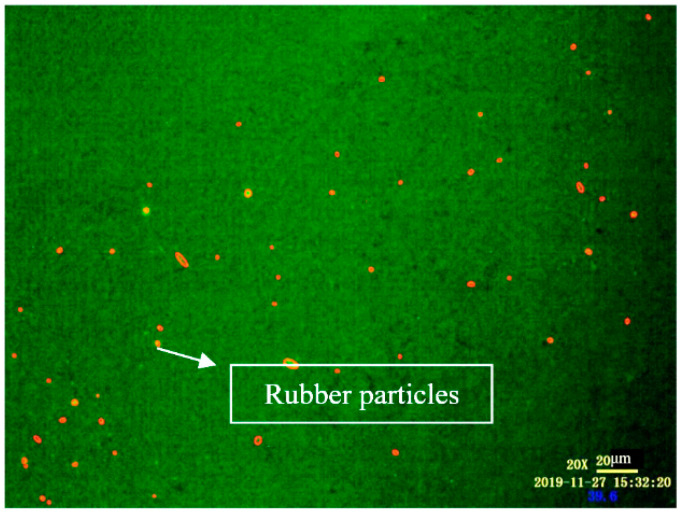
FM images of disintegrated high volume crumb rubber asphalt (DHVRA) labeled with area of interest (AOI).

Selection of AOI: Due to the low brightness, low definition and irregular shape of the CR particles in FM, it is not easy to be selected. Therefore, CR particles in the images were marked with a red circle by the manual marking method. The marked image is shown in Figure 2.

Indicators calculation: The max diameter, min diameter and mean diameter were calculated by the software. Ten FM pictures were taken of different RA, and the average value was calculated as the final result.

#### 2.3.2. Analysis of Disintegrating Mechanism of DHVRA

##### Infrared Spectroscopy (IR)

The Varian 600-IR Fourier infrared spectroscopy (FT-IR) was used. The test range of FT-IR was 4000–600 cm^−1^. The attenuated total reflection (ATR) sample preparation method is to put the asphalt sample directly on the ATR crystal and use the principle of total reflection of light to get the IR of asphalt. This method has the advantages of fast scanning, less sample quantity and no damage to the samples, etc., and is more suitable for RA. Therefore, in this paper, ATR sample preparation method was selected to carry out the FT-IR test of HVRA, and the OMNIC software was used for the pretreatment of IR.

##### Gel Permeation Chromatography (GPC)

The PL-GPC 50 Gel chromatograph was selected to measure different asphalt samples. The elution time of polymer with different molecular size passing the chromatographic column differed, so as to distinguish the substances with different molecular weight and measure the molecular size and molecular weight distribution. The flowing phase was tetrahydrofuran (THF), the flowing rate of the instrument was 0.1 mL/min, the sample concentration was 1 mg/mL, the test temperature was 25 °C and the injection volume was 100 μL. Gel Permeation Chromatography (GPC) can obtain the average molecular weights. Numerical average molar weight *M_n_* and weight-average molar weight *M_w_* are commonly used, and the equations are shown as Equations (3) and (4).

(3) $$M_{n}=\sum N_{i}\cdot M_{i}/\sum N_{i}=\sum W_{i}\cdot M_{i-1}$$

(4) $$M_{w}=\sum W_{i}\cdot M_{i}/\sum W_{i}$$

where *N_i_* is the number of molecules with a molecular weight of *M_i_*; *W_i_* is the weight of component with molecular weight *M_i_*.

##### Contact Angle Tests and Determination of Surface Free Energy

The surface free energy (SFE) of different asphalts was determined indirectly by the sessile drop method. The contact angle measuring instrument was a video optical contact angle instrument produced by the German Data Physics company. The solid asphalt membrane used to measure the contact angle was made by inserting the glass sheet into the molten asphalt, then hanging it upside down in the oven for 12 h. In order to ensure the reliability of the test, three parallel samples were prepared for each asphalt. The measured liquid was distilled water and ethylene glycol, and the test temperature was 25 °C.

The SFE of liquid and solid can be calculated according to Equations (5) and (6), respectively.

(5) $$\gamma_{l}=\gamma_{l}^{d}+\gamma_{l}^{p}$$

(6) $$\gamma_{s}=\gamma_{s}^{d}+\gamma_{s}^{p}$$

where $\gamma_{l}^{d}$ and $\gamma_{l}^{p}$ are dispersion and polarity components of liquid SFE, respectively; $\gamma_{s}^{d}$ and $\gamma_{s}^{p}$ are dispersion and polarity components of solid SFE; *γ_l_* is SFE of liquid; *γ_s_* is SFE of solid.

Based on Equation (7), the SFE of solid–liquid interface can be calculated, and the relationship between the contact angle of the liquid on solid and the SFE is shown in Equation (8).

(7) $$\gamma_{sl}=\gamma_{s}+\gamma_{l}-2\sqrt{\gamma_{s}^{d}\gamma_{l}^{d}}-2\sqrt{\gamma_{s}^{p}\gamma_{l}^{p}}$$

(8) $$\gamma_{l}\cos\theta =\gamma_{s}-\gamma_{sl}$$

Combining Equations (7) and (8), the Equation (9) between the contact angle *θ* and the SFE of solid is obtained [37].

(9) $$1+\cos\theta =2\sqrt{\gamma_{s}^{d}}\left(\sqrt{\gamma_{l}^{d}}/\gamma_{l}\right)+2\sqrt{\gamma_{s}^{p}}\left(\sqrt{\gamma_{l}^{p}}/\gamma_{l}\right)$$

Since the $\gamma_{l}^{d}$ and $\gamma_{l}^{p}$ of distilled water and glycol are known, as long as the contact angle between two liquids and asphalt materials was determined separately, $\gamma_{s}^{d}$ and $\gamma_{s}^{p}$ of each solid asphalt can be calculated by Equation (9).

#### 2.3.3. Rheological Properties of DHVRA

MCR 302 Dynamic Shear Rheometer (DSR) manufactured by Anton Paar Company was used to evaluate the rheological performance of different asphalt binders.

##### Temperature Sweep

The temperature was tested in the range of 46–82 °C by intervals of 6 °C. The test frequency was 10 Hz, and the strain was 10%. The parallel plate diameter was 25 mm, and the gap between parallel plates was 2 mm. According to the high-temperature sweep test, the parameters of complex shear modulus G*, phase angle *δ*, complex viscosity and rut factor G*/sin*δ* could be obtained.

##### Low-Temperature Frequency Sweep (FS)

The test methods of the Stratagem Highway Researching Plan (SHRP ) to evaluate the low-temperature performance of asphalt binder are bending beam rheometer (BBR) and direct tension test (DTT). However, due to the large amount of time consumed in the BBR test, the 4 mm parallel plate mold was introduced and used a frequency sweep (FS) test to test asphalt binder at low temperature [38,39]. Compared to the creep stiffness modulus *S(t)* master curve obtained by the BBR test with relaxation modulus *G(t)* master curve obtained by FS test, the correlation coefficient between *G* (60 s) and *S* (60 s) was 0.85. Therefore, this paper used the FS test instead of the BBR test to evaluate the low-temperature performance of rubber asphalt, and the test temperature was −12 °C.

## 3. Results and Discussions

### 3.1. Determination of Disintegrating Agent (DA) Content

**Figure 3:**
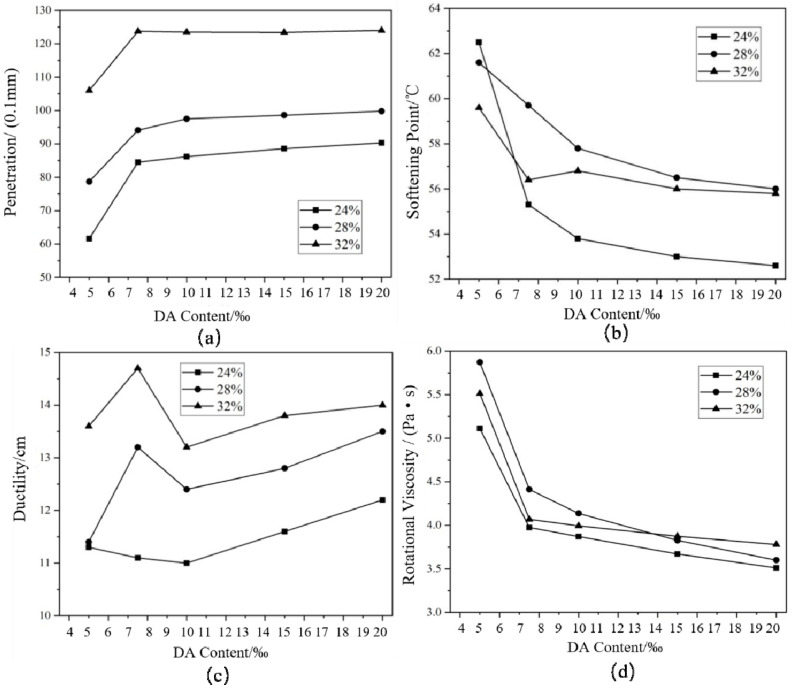
Conventional technical indexes of DHVRA with different disintegrating agents (DAs): (**a**) penetration; (**b**) softening point; (**c**) ductility; (**d**) rotational viscosity.

It can be seen in Figure 3 that with the increase in CR content, the penetration and ductility of RA increased, while the change rule of softening point and RV was not significant. Moreover, with the increase in DA content, the penetration and ductility increased, and the softening point and RV decreased. As the DA dosage increased from 0.5% to 7.5‰, the conventional index of HVRA changed significantly. When DA content exceeded 7.5‰, the relevant indexes still changed, but the change range was very narrow. At this time, the increase in the amount of DA did not obviously increase the cracking effect of CR. It indicated that when the DA content was 7.5‰, it was the most cost-effective. Considering that the increase of DA would also increase the production cost of HVRA, a DA content of 7.5‰ was determined in this study.

### 3.2. Analysis of Technical Characteristics of DHVRA

#### 3.2.1. Analysis of Key Indicators of Long-Term Disintegrating Tests

##### Penetration

**Figure 4:**
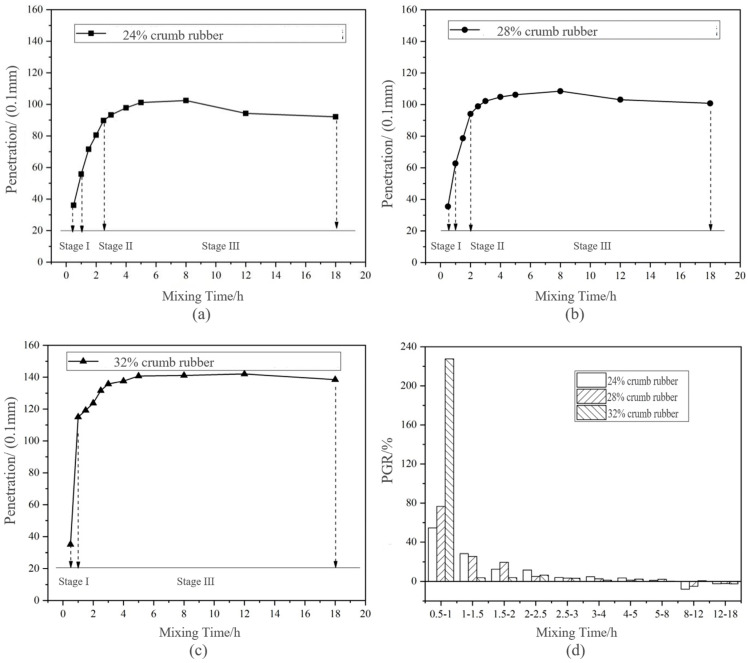
Change of penetration and penetration growth rate (PGR) of DHVRA with mixing time increasing based on long-term disintegrating: (**a**) 24% crumb rubber (CR); (**b**) 28% CR; (**c**) 32% CR; (**d**) PGR with three CR contents.

During the long-term disintegrating test, the variation of penetration is shown in Figure 4. Based on the change of PGR in Figure 3, the disintegrating of DHVRA was roughly divided into three stages. The corresponding mixing time and PGR are shown in Table 5.

Stage I: The PGR in this stage was above 50%, and the higher the CR content was, the larger the PGR was. Combining this with Figure 4, it can be concluded that the PGR of the HVRA with the content of 32% CR reached about 220% during mixing for 0.5–1 h. This was much higher than that of the other two RA with the same mixing time. It showed that with the increase of CR content, at the initial of mixing (0.5–1 h), the PGR of RA increased, the penetration increased faster, the disintegrating product increased, and the disintegrating reaction of CR was more violent. Because the largest PGR was in this stage, it was named rapid disintegrating stage.

Stage II: In this stage, the PGR was lower than that of stage I and was stable in 10–30%, so it was called the stable disintegrating stage. According to Figure 3, with the increasing CR content, the duration of the stable disintegrating stage was shorter. However, based on the PGR, there was no stage II of DHVRA with 32% CR. This may be due to the insensitivity of the penetration index to the disintegrating time increasing, when the CR content was 32%. 

Stage III: The PGR in this stage was less than 5%, and the penetration curves of DHVRA under different CR contents were generally gentle. This indicated that the disintegrating reaction in this stage was relatively slow, so this stage was called the micro-disintegrating stage. From Figure 3, the PGRs of the DHVRA with 24% and 28% CR content were negative when the mixing time was 8–18 h, whereas that with 32% CR content was negative when the mixing time was 12–18 h. This showed that penetration of the three kinds of DHVRA after mixing for 8 h had negative growth, and the RA began to age, and the higher the CR content, the corresponding mixing time of appearing aging was longer. The main reason was that the greater the amount of CR, the more carbon black in RA. The presence of some anti-aging agents in carbon black and CR improved the aging resistance of RA. Therefore, the mixing time should be controlled within 8 h to ensure the RA is not aged.

Combining Table 5 with Figure 4, it can be concluded that the disintegrating process was divided into three stages: rapid disintegrating stage, stable disintegrating stage and micro-disintegrating stage. The fast disintegrating stage and the stable disintegrating stage were not long, and the micro-disintegrating stage lasted the longest at about 1–2 h. Furthermore, the mixing time of the disintegrating stage was different with different CR content. The higher the CR content, the greater the PGR in the rapid disintegrating stage, the shorter the duration of the stable disintegrating stage and the longer of micro-disintegrating stage.

##### Rotational Viscosity (RV)

**Figure 5:**
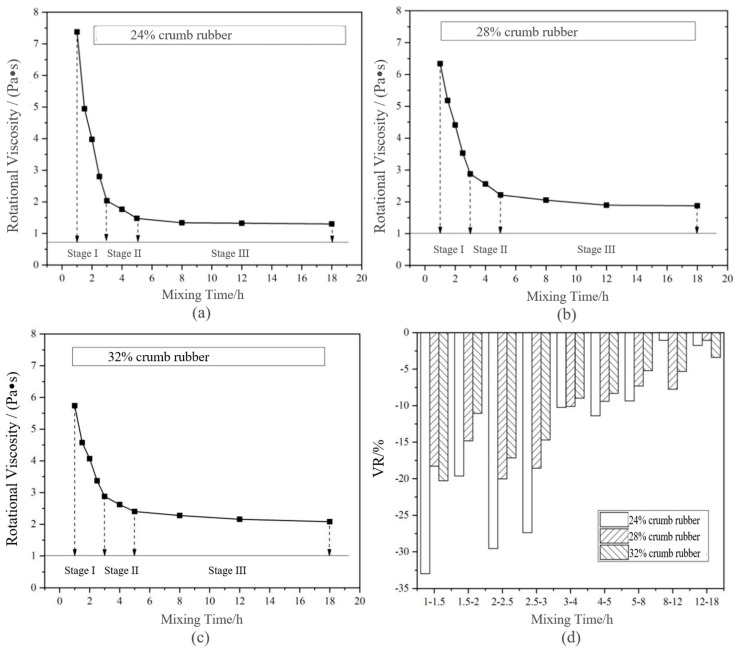
Change of rotational viscosity (RV) and viscosity decay rate (VDR) of DHVRA with mixing time increasing based on long-term disintegrating: (**a**) 24% CR; (**b**) 28% CR; (**c**) 32% CR; (**d**) VDR with different CRs.

Figure 5 shows that the 135 °C RV changed with the mixing time under long-term disintegrating. Based on VDR, the long-term disintegrating phase division is shown in Table 6.

Combining Figure 5 and Table 6, it can be concluded that, similar to the PGR, the long-term disintegrating of DHVRA can be roughly divided into three stages based on the change of VDR. The differences between the two criteria are as follows: (1) the mixing time of the disintegrating stage based on PGR was different, while the mixing time of the disintegrating stage based on VDR was the same. It can be seen that the stage division based on the VDR index was more generalizd. (2) When the content of CR was 32% and the mixing time was 2–3 h, the PGR of rubber asphalt was less than 10%, while the VDR was still 20–30%. Therefore, it can be considered that the sensitivity of RV to disintegrating change of HCRA was higher than penetration.

Because the mixing time of each disintegrating stage divided by penetration and RV was not consistent, to select the most suitable indicators to evaluate the technical characteristics of DHVRA, the correlation analysis of penetration, RV and other technical indexes of DHVRA should be carried out.

#### 3.2.2. Storage Stability

As shown in Table 7, the softening point differences of three RAs increased with the extension of storage time, which indicated that the segregation of RAs was more serious with increasing storage time. Under the same storage time, the difference between the upper and lower softening points of DHVRA is smaller than that of CRA. Moreover, compared with DHVRA mixed for 2 h, the softening point difference with mixing for 3 h was smaller. This showed that the addition of DA could significantly improve the segregation of RA, and with the extension of mixing time, the improvement effect was better. As a result, the addition of DA could better improve the storage stability of RA.

#### 3.2.3. Fluorescence Microscope (FM) 

##### Fluorescence Microscope (FM) Images

**Figure 6:**
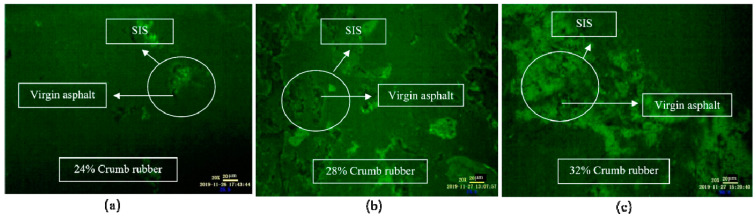
FM images of DHVRA with mixing for 1 h: (**a**) 24% CR; (**b**) 28% CR; (**c**) 32% CR.

**Figure 7:**
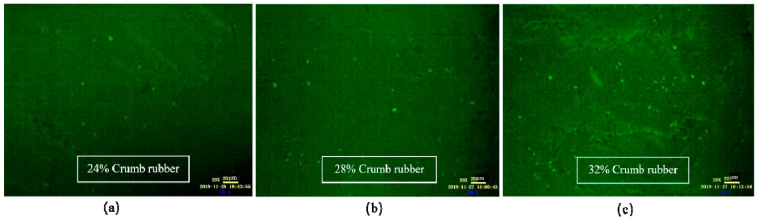
FM images of DHVRA with mixing for 3 h: (**a**) 24% CR; (**b**) 28% CR; (**c**) 32% CR.

**Figure 8:**
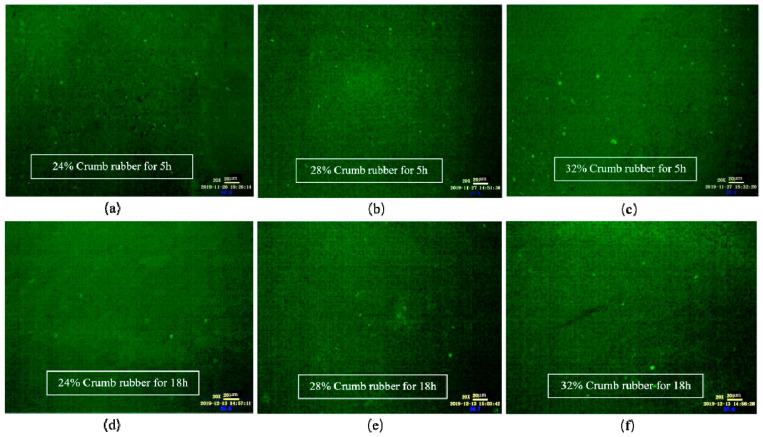
FM images of DHVRA with mixing for 5 h and 18 h: (**a**) 24% CR, 5 h; (**b**) 28% CR, 5 h; (**c**) 32% CR, 5 h; (**d**) 24% CR, 18 h; (**e**) 28% CR, 18 h; (**f**) 32% CR, 18 h.

As shown in Figure 6, when the mixing time was 1 h, the CR absorbed the light component in VA rapidly and swelled, and a large flocculent “sea island structure (SIS)” was formed and dispersed in the whole reaction system. In FM images, the yellow part shows the swelling rubber particles, and the black part shows VA. Moreover, the higher the CR content was, the larger the SIS volume was. According to Figure 7, after mixing for 3 h, the 3D network structure of CR was destroyed, and the SIS was gradually decomposed into smaller particles and distributed in the reaction system. As shown in Figure 8, when the mixing time was 5 h, the number of CR particles decreased obviously and the size further decreased. After mixing for 18 h, the CR particles could still be observed in the FM images, and the RA was heterogeneous in structure. This showed that the disintegrating reaction of CR was always kept running in the preparation process, and it was difficult to completely dissolve the CR in base asphalt by heating and mixing.

##### Analysis of FM Images Based on Image-Pro Plus (IPP)

When the mixing time was less than 2 h, the SIS was irregular and difficult to measure. Until the mixing time was greater than 2 h, the SIS could be basically decomposed into small granular structure and measured by IPP. Therefore, the FM images of DHVRA after mixing for 2 h were selected as the measurement objects. 

**Figure 9:**
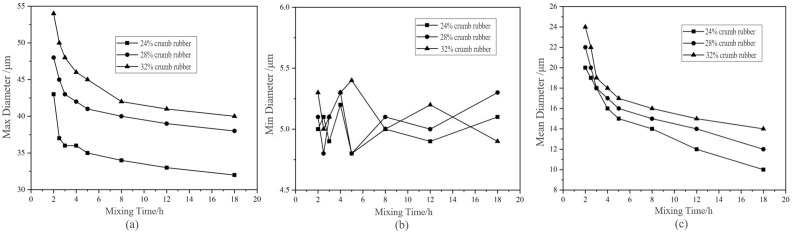
Change curves of particle diameter of DHVRA with mixing time increasing. (**a**) Max diameter; (**b**)Min diameter; (c) Mean diameter

The results shown in Figure 9 reflect that with the increase of mixing time, the CR particle size decreased gradually, and the max diameter and mean diameter of CR particles also decreased. The higher the CR content, the larger the max diameter and mean diameter of CR particles. However, the change in the min particle size of CR was not obvious. Additionally, it was different from the changes in penetration and RV, the characteristic indexes of DHVRA mostly no longer changed after mixing for more than 8 h, but at the micro state, the CR particles continued decreasing, and the disintegrating reaction kept running.

#### 3.2.4. Correlation Analysis of Evaluation Indexes

In this paper, the linear fitting between penetration, 135 °C RVand particle diameter of CR was carried out to obtain the most appropriate key index between penetration and RV.

**Figure 10:**
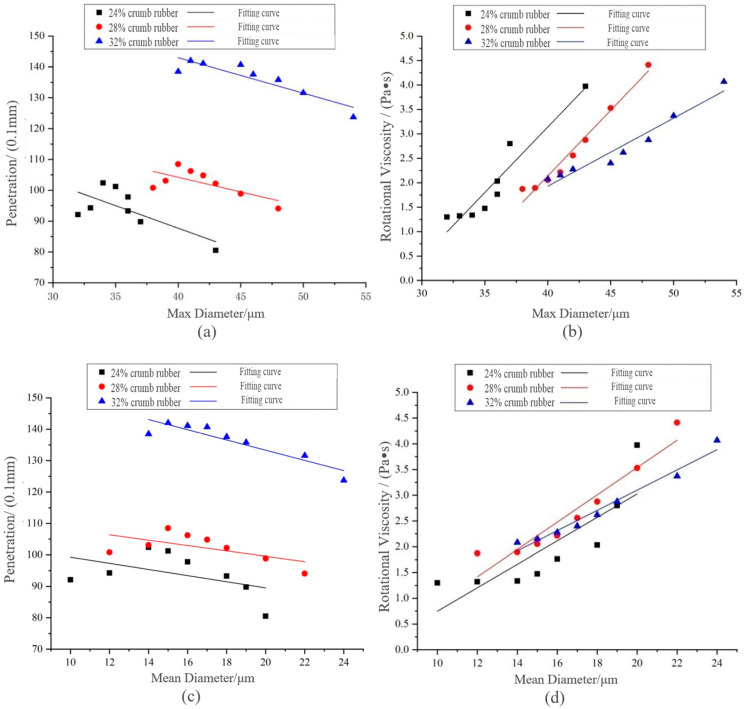
Linear fitting between penetration, RV and CR particle size of DHVRA: (**a**) penetration and max diameter; (**b**) RV and max diameter; (**c**) penetration and mean diameter; (**d**) RV and mean diameter.

Combining Figure 10 with Table 8, it can be deduced that when CR contents were 24% and 28%, the correlation coefficients R^2^ between penetration and max diameter and mean diameter were less than 0.45, whereas R^2^ between RV and max diameter and mean diameter was above 0.7. Therefore, penetration index could not adequately explain the diameter change of CR in the preparation process. The RV was closely related to the disintegrating characteristics and could be used as a key evaluation index of DHVRA. Therefore, RV at 135 °C was selected as the key evaluation index of DHVRA, and VDR was recommended for the division standard of the disintegrating stage. Considering that the longer the disintegrating time was, the more the high-temperature performance of DHVRA decreased, the higher the risk of asphalt aging, and with a higher preparation cost of asphalt, the preparation time is expected not to be too long. Since the key index of DHVRA was 135 °C RV, for identifying it quickly and accurately in practical engineering applications, the DHVRA with 3 h mixing time is recommended for the follow-up study and is called DRA for short. Moreover, the 135 °C RV of DRA in the range of 1.475–2.876 Pa∙s was proposed.

### 3.3. Analysis of Disintegrating Mechanism of DHVRA

The names of different asphalt were simplified as shown in Table 9.

#### 3.3.1. FT-IR

##### FT-IR Spectrums of Different Asphalt

**Figure 11:**
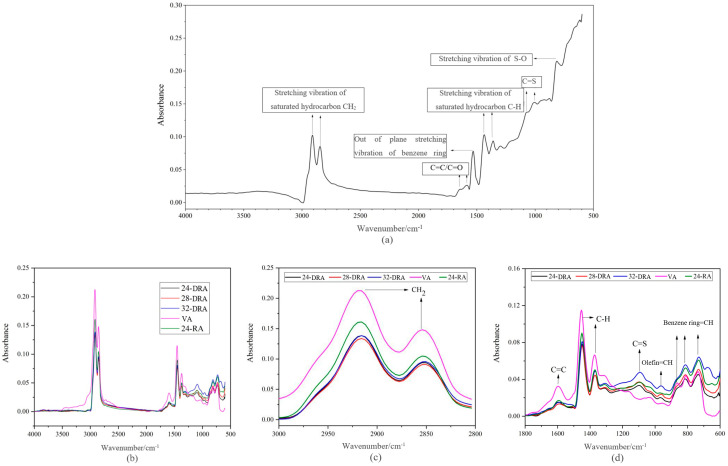
FT-IR spectrogram of CR and different asphalt binders: (**a**) CR; (**b**) FT-IR spectrum; (**c**) absorption peak; (**d**) functional groups at absorption peak.

Figure 11 shows the FT-IR spectrums of CR, 24-DRA, 28-DRA, 32-DRA, 24-RA and VA. It can be found from Figure 11b that the differences in absorption peaks among these asphalts were mainly in the wavenumber of 3000–2800 cm^−1^ and lower than 1800 cm^−1^ [40]. The two parts of the spectrum were selected separately and are shown in Figure 11c,d.

It can be deduced from Figure 11b,c that there were broad absorption peaks at 2846 cm^−1^ and 2911 cm^−1^ caused by stretching vibration of saturated hydrocarbon CH_2_. There was a broad absorption peak at about 1600 cm^−1^ due to the vibration of the conjugated C=C double bond of the benzene ring, and a sharp absorption peak caused by stretching vibration of the C-H bond of saturated alkanes at 1450 cm^−1^ and 1370 cm^−1^. Moreover, the three kinds of absorption peaks of VA were obviously higher than that of RA. As seen in Figure 11a, the absorption peaks caused by out-of-plane vibration of benzene ring CH appeared at about 1529 cm^−^^1^, and the absorption peak caused by C=C (vibration skeleton of benzene ring) and C=O appeared at about 1600 cm^−^^1^, which indicated that aromatic compounds existed in CR. Combined with Figure 11b, peak area coming from CH_2_, C-H and C=C groups of VA was larger than that of CR, which means the content of saturated hydrocarbon and C=C (C=O) bond in the CR was lower than that of VA. Therefore, when a large amount of CR was added in the preparation process, a part of the base asphalt was replaced by CR, and the content of base asphalt decreased in the same volume of the asphalt binder system. As a result, the content of saturated hydrocarbon and C=C (C=O) bond in CR was lower than that in VA. Therefore, the content of saturated hydrocarbon and C=C (C=O) bond would decrease during the preparation of RA. In addition, compared with VA, RA showed a medium-strength absorption peak caused by C=S bond stretching at 1100 cm^−1^. This was due to the fact that there were a large number of C=S bonds in the CR. Furthermore, there were three absorption peaks in the benzene ring substitution region 870–730 cm^−1^ caused by the vibration of benzene ring =C-H, while RA had only two absorption peaks in that region. The reduction of the absorption peak in the benzene ring substitution zone indicated that the chemical reaction occurred in the preparation process of RA. Compared with VA and 24-RA, DRA had a weak absorption peak caused by the out-of-plane bending of trans olefin =C-H in 960 cm^−1^. The formation of a new absorption peak in DRA may be caused by some components of DA added in DRA, and new functional groups were produced in the disintegrating process of HVRA. 

##### FT-IR Spectrum of Different DHVRA

Comparing the Spectrum of 2405, 24-DRA, 2805, 28-DRA, 3205 and 32-DRA, it is found that the absorption peaks of these asphalts were different from those of 3000–2800 cm^−1^ and lower than 1800 cm^−1^. The spectrums of these two parts were selected separately. 

**Figure 12:**
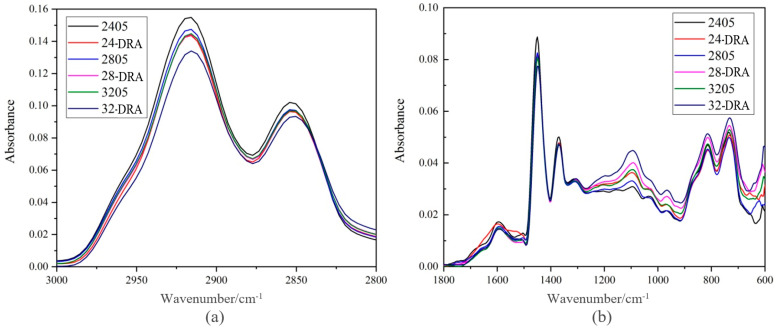
Absorption peaks of DHVRA: (**a**) absorption peaks at 3000–2800 cm^−1^; (**b**) absorption peak at 1800 cm^−1^.

Figure 12 shows the absorption peak of asphalt spectrum of different DHVRAs. From Figure 11, with the increase in mixing time, the absorbance of the absorption peak at 3000–2800 cm^−1^ decreased, whereas that at 1200–900 cm^−1^ increased. The absorbance of the absorption peak at other positions did not change significantly. The functional group corresponding to the absorption peak at 3000–2800 cm^−1^ was CH_2_, and the corresponding functional group at 1200–900 cm^−1^ was olefin =C-H and C=S. This showed that the functional groups in VA reacted with some components in CR during the mixing process. Moreover, due to the decomposition of rubber hydrocarbon, the saturated hydrocarbon content in DHVRA decreased and the olefin content increased.

#### 3.3.2. GPC

**Figure 13:**
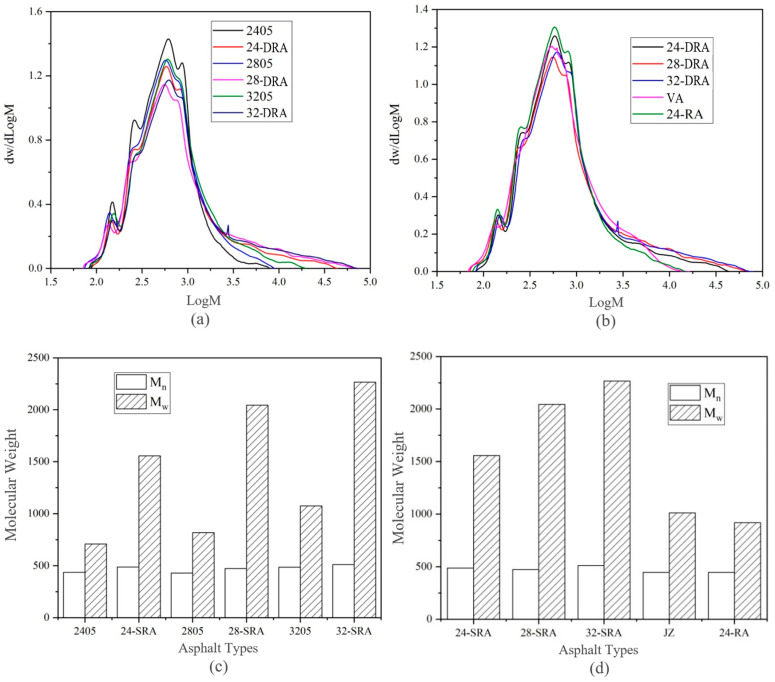
Molecular weight of different asphalt binders: (**a**,**b**), molecular weight distribution; (**c**,**d**), molecular weight.

To study the influence of mixing time on the molecular weight, the asphalt sample was dissolved in THF, the mixed solution was filtered and injected into the equal volume ring with a syringe. The molecular weight distribution of different asphalt was obtained by GPC test, as shown in Figure 13.

According to Figure 13a, the molecular weight of DHVRA in the rapid disintegrating stage was 10^1.8^–10^4.3^, while that in the stable disintegrating stage was in the range of 10^1.9^–10^4.8^ and was slightly higher than that in the rapid disintegrating stage. This shows that the disintegrating of CR made the high molecular weight of RA increase, while the small molecular weight was basically unchanged. Moreover, the GPC curves of DHVRA as a whole moved to the right, and the higher the CR content, the more the curve moved. Additionally, from Figure 13b, the molecular weights of VR and 24-RA were similar and changed from 10^1.8^ to 10^4.2^, while the molecular weights of DRA were significantly higher than those of the two kinds of asphalt. The main reason was that the pyrolysis reaction of CR and the reaction between CR and VA in 24-RA were relatively slight, while those reactions in DRA were relatively vigorous. Therefore, the molecular weights of 24-RA and VA were at the same level and were significantly lower than those of DRA.

It can be concluded from Figure 13c,d that the number average molecular weight M_n_ of DRA was slightly higher than that of other asphalts. The main difference was that the *M_w_* of DRA was 1.5–2.2 times that of 24-RA and VA, and the higher the CR content, the higher the *M_w_* of DRA. This is because, during the mixing process of DHVRA, macromolecular substances are decomposed from CR and infiltrated into VA. At the same time, the light components of the VA could volatilize during the high-temperature mixing process, which made the *M_w_* in DRA increased significantly [41].

#### 3.3.3. Contact Angle Test and Determination of Surface Free Energy (SFE)

Based on the contact angle test results of different asphalts with distilled water and glycol (shown in Table 10), and combined with Equation (9), the SFE of different asphalts was calculated and is shown in Table 11. 

Table 11 indicates that the polar component of VA and 24-DRA accounted for a small proportion, so both VA and 24-DRA presented very weak polarity. In addition, with the increase in CR content, the polar component of 28-DRA and 32-DRA increased. Compared with the SFE of 24-RA, the polar component of the DRA was higher, which indicates that the addition of DA accelerated the disintegrating process of CR, and rubber hydrocarbon in CR was cracked out [42]. Therefore, the polar component of RA surface energy increased. Furthermore, when the amount of CR was constant, the polar component of DHVRA increased with the increase in mixing time. This was because, with the extension of mixing time, the rubber hydrocarbon in CR was disintegrated into the VA. Asphalt is mainly composed of non-polar hydrocarbons, and its dispersion component accounts for the main part of surface energy [43]. With the addition of DA, CR absorbed the more non-polar light oil components in base asphalt and transformed them into resins and asphaltenes with larger polarity [33]. These resins have high polarity because they contain fused ring and heterocyclic compounds, which generates high surface free energy. More resins in asphalt will result in higher surface free energy of the asphalt sample [43]. According to the surface energy theory, the adhesion work is closely related to the polarity of two or more phases when they are bonded or wetted [44]. The polarity component of DHVRA increased, and the adhesion work generated by the polarity component was also greater. Therefore, DHVRA had better adhesion performance with aggregate.

### 3.4. Analysis of High-Temperature Rheology Properties of DHVRA

#### 3.4.1. Indicators of Rheology Properties

**Figure 14:**
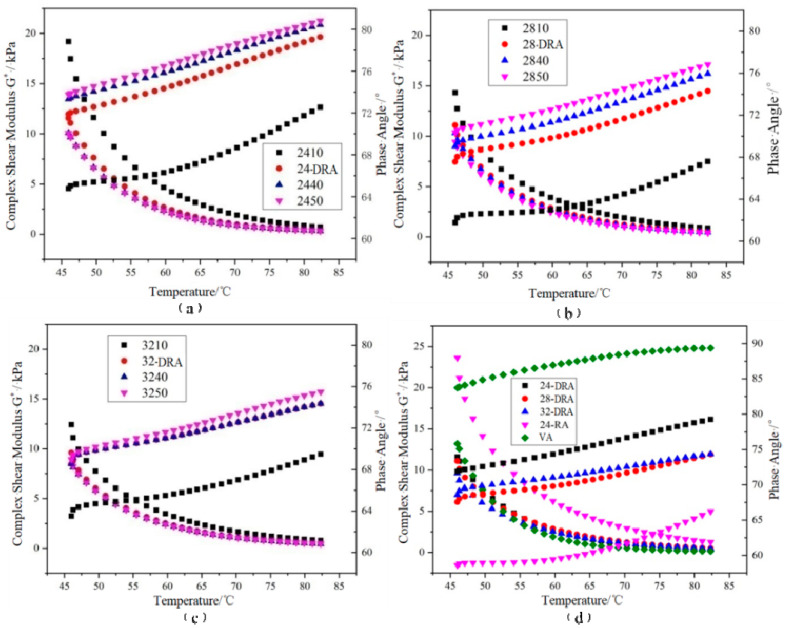
Complex shear modulus G* and phase angle *δ* curves of different asphalt binders: (**a**) 24% CR; (**b**) 28% CR; (**c**) 32% CR; (**d**) five asphalt binders.

**Figure 15:**
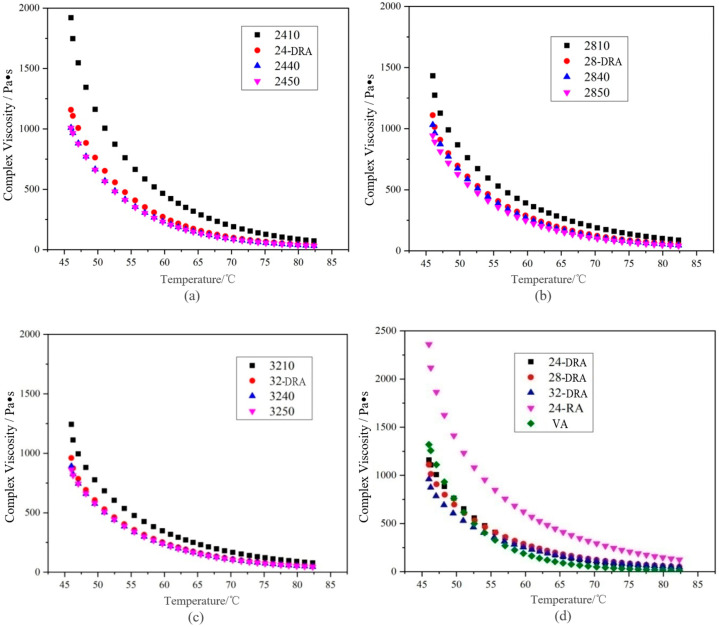
Complex viscosity curves of different asphalt binders: (**a**) 24% CR; (**b**) 28% CR; (**c**) 32% CR; (**d**) five asphalt binders.

**Figure 16:**
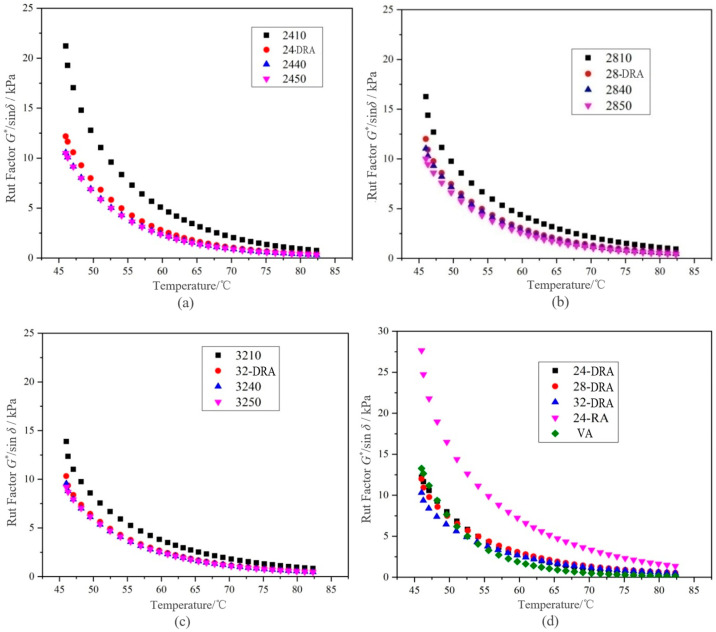
Rut factors curves of different asphalt binders: (**a**) 24% CR; (**b**) 28% CR; (**c**) 32% CR; (**d**) five asphalt binders.

It can be deduced from Figure 14, Figure 15 and Figure 16 that with the mixing time increasing, the complex shear modulus G*, complex viscosity and rut factor decreased, and the flow deformation resistance of DHVRA decreased. The phase angle *δ* was in proportion to the mixing time, and the longer the mixing time, the less the elastic components and the more plastic components in RA system. After mixing for 1 h, with the increase in CR content, both of the complex shear modulus G* and phase angle δ decreased, and the complex viscosity and rut factor were inversely proportional to CR content. Furthermore, in the stable disintegrating stage, the complex shear modulus G* of DHVRA was similar to VA, the phase angle *δ* was between 24-RA and VA, and the complex viscosity and rut factor fluctuated within a narrow range. With the CR content changing, the viscoelastic parameters of RA had little difference. With the progress of the disintegrating reaction, the viscoelastic parameters of DHVRA were closer and closer to the VA. In particular, high-temperature deformation resistance of DHVRA decreased, elastic composition was reduced and composite viscosity declined. The variation of viscoelastic parameters with mixing time was in accordance with the change rules that penetration and RV were directly and inversely proportional to mixing time, respectively.

#### 3.4.2. Temperature Sensitivity and Failure Temperature Analysis

**Figure 17:**
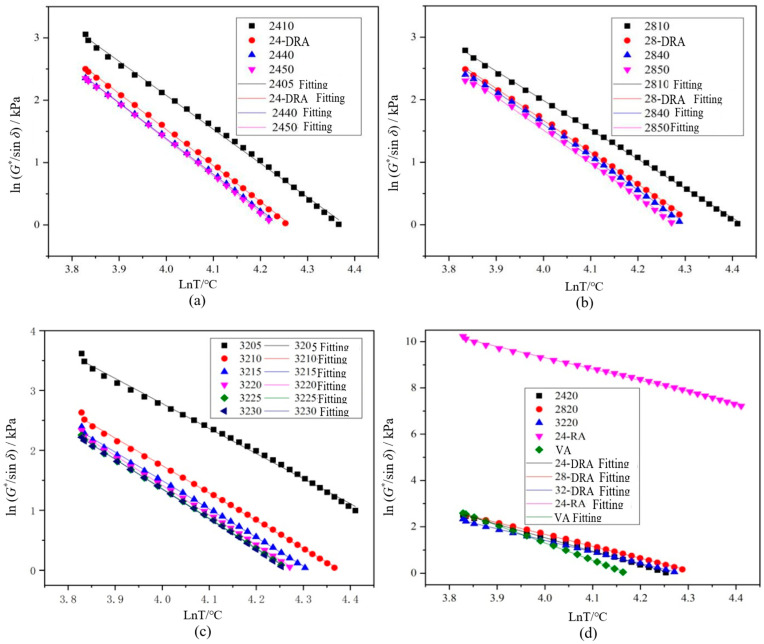
Rut factors fitting curves of different asphalt binders: (**a**) 24% CR; (**b**) 28% CR; (**c**) 32% CR; (**d**) five asphalt binders.

The correlation between rut factor and rut resistance of asphalt pavement was very low [45]. The data of rut factor G*/sin*δ* less than 1.0 kPa were eliminated, and the remaining data were calculated by logarithm. The linear fitting curves based on shear temperature are shown in Figure 17. The function relationships and evaluation indexes obtained by linear fitting of rut factor and shear temperature under semi-logarithmic coordinates are shown in Table 11.

According to Table 12, it can be concluded that with the extension of mixing time, the temperature sensitivity index of DHVRA increased. As a result, the longer the mixing time, the worse the temperature sensitivity of RA. In addition, when the mixing time was constant, the temperature sensitivity index of DHVRA decreased with the increase in CR content, so that the higher the rubber powder content, the better the temperature sensitivity of RA. Furthermore, when the amount of CR was constant, the failure temperature decreased with the increase in mixing time, and while the mixing time was fixed, the order of failure temperature was 28-DRA > 32-DRA > 24-DRA. Therefore, the high-temperature deformation resistance of asphalt was 28-DRA > 32-DRA> 24-DRA.

To sum up, it was not the case that the greater the CR content of DHVRA the better, and 28% CR content is recommended for field engineering.

### 3.5. Analysis of Low-Temperature Rheology Properties of DHVRA

The angular frequency *ω*, storage modulus *G′* and loss modulus of asphalt can be obtained by frequency sweep (FS). There is a conversion equation between the storage modulus master curve *G′*(*ω*) and relaxation modulus master curve *G*(*t*). Among many transformation equations, the one proposed by Christensen [46] (as shown in Equation (10)) is widely accepted for its high accuracy. Therefore, in this paper, Equation (10) was used to fit the master curve.

(10) $$G\left(t\right)\approx {G^{\prime }\left(\omega \right)|}_{\omega =2/\pi t}$$

When the relaxation modulus master curve *G*(*t*) was obtained, the relaxation modulus *G′* (60 s) and relaxation rate *m_c_* (60 s) of asphalt sample after 60 s relaxation were calculated according to Equations (11) and (12).

(11) $$\mathrm{lg}G\left(60s\right)=ax^{2}+bx+{c|}_{x=\log\left(60\right)=1.78}$$

(12) $$m_{r}\left(60s\right)=2ax+{b|}_{x=\mathrm{lg}\left(60\right)=1.78}$$

**Figure 18:**
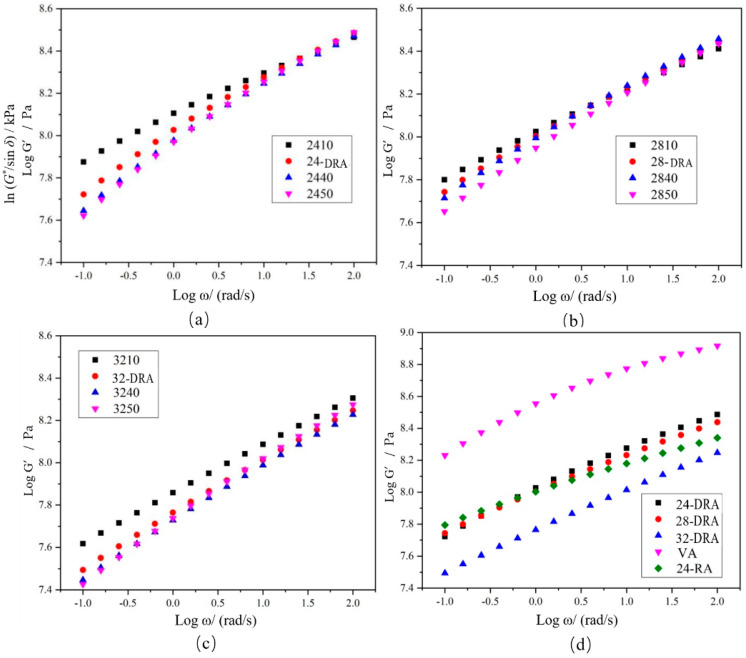
Master curve of storage modulus *G′*: (**a**) 24% CR; (**b**) 28% CR; (**c**) 32% CR; (**d**) three DRAs, VA and 24-RA.

**Figure 19:**
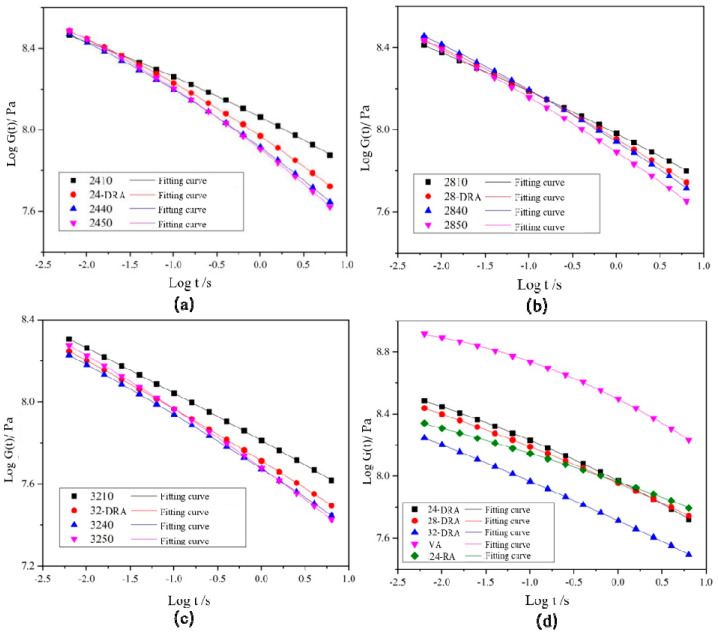
Master curve of relaxation modulus *G*: (**a**) 24% CR; (**b**) 28% CR; (**c**) 32% CR; (**d**) three DRAs, VA and 24-RA.

**Figure 20:**
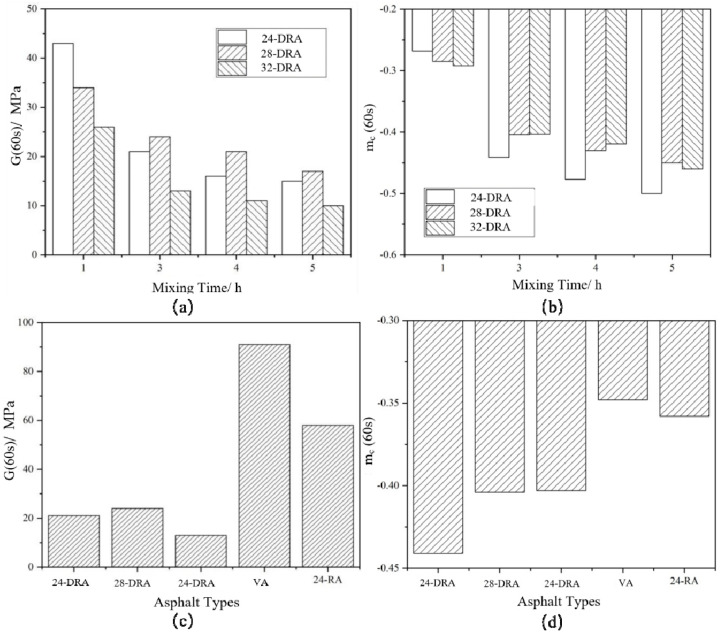
Relaxation modulus *G* (60 s) and relaxation rate *m_c_* (60 s) of different asphalts: (**a**) *G* (60 s) of DRA; (**b**) *m_c_* (60 s) of DRA; (**c**) *G* (60 s) of three DRAs, VA and 24-RA; *(***d***) m_c_* (60 s) of three DRAs, VA and 24-RA.

Logarithms of storage modulus *G′* and angular frequency *ω* were taken to obtain the master curves of the storage modulus of each asphalt binder, as shown in Figure 18. The FS data were converted and then were fitted by quadratic polynomial to obtain the master curve of relaxation modulus *Log G(t)−Log t* of each asphalt binder, and the results are shown in Figure 19. Finally, the relaxation modulus *G* (60 s) and relaxation rate *m_c_* (60 s) were calculated. *G* (60 s) and *m_c_* (60 s) were the relaxation modulus and relaxation rate at 60 s, respectively, obtained by data fitting. The *G* (60 s) and *m_c_* (60 s) of different asphalt binders are shown in Figure 20. It can be found from Figure 18, Figure 19 and Figure 20 that the low-temperature relaxation ability of DHVRA was improved with the extension of mixing time. When CR content was constant, *G* (60 s) decreased rapidly with the increase in mixing time. However, when asphalt reached a stable disintegrating stage, *G* (60 s) decreased slowly. Moreover, the absolute value of relaxation rate *m_c_* (60 s) increased rapidly when DHVRA transitioned from the rapid disintegrating stage to stable disintegrating stage, whereas *m_c_* (60 s) increased slowly as asphalt reached the stable disintegrating stage. In general, the low-temperature relaxation energy of DRA was better than that of 24-RA and VA, and the order of relaxation modulus of three DRAs with different CR content was: 32-DRA < 24-DRA < 28-DRA. With the increase in CR content, the relaxation rate decreased and the low-temperature relaxation ability was improved. Hence, 32-DRA had the best relaxation ability at low temperature.

## 4. Conclusions

The traditional production method of RA has a high cost and low efficiency and increases the probability of environmental pollution, and the products have defects such as high viscosity, easy agglomeration and poor low-temperature performance. In order to maximize the utilization of waste CR, obtain the homogeneous RA with low viscosity and good storage stability and solve the poor low-temperature performance at the same time, the DHVRA is proposed. By determining the optimum evaluation indexes, analyzing the micro-disintegrating mechanism, and evaluating the high- and low-temperature rheological properties, a more comprehensive and profound understanding of the performance of DHVRA was obtained, and the following conclusions can be drawn:

(1) Through the analysis of preparation parameters and main technical indicators, the optimal DA dosage of 7.5‰ and the mixing time of 3 h are proposed, and 135 °C RV is suggested as the optimum index to evaluate the disintegration characteristics. Combining the high- and low-temperature rheological properties, the CR content of 28% is recommended for high-temperature areas, and the 32% CR is more suitable for low-temperature. 

(2) An RV of 135 °C is proposed as the best key index. Based on the viscosity decay rate (VDR), the disintegrating process is divided into three stages. Especially in the fast disintegrating stage (mixed for 0–3 h), the FM images mainly consist of a large irregular “SIS”. The 135 °C RV decreases, and workability is improved markedly, and the DHVRA with 135 °C RV in the range of 1.475–2.876 pa·s is recommended. After mixing for 3 h, all the indicators change slightly. After 8 h, the asphalt aging occurs. It should be noticed that due to the presence of DA, the particle size of CR continues decreasing, and the disintegrating reaction is kept running.

(3) Compared to VA and 24-RA, there is a weak absorption peak in DHVRA infrared spectrum at 960 cm^−1^ caused by trans olefin =C-H, and with the increase in mixing time, the content of C=S bond and olefin =C-H bond increases. Moreover, the molecular weight of DHVRA is higher, the *M_w_* of DHVRA was 1.5–2.2 times that of 24-RA and VA, and the higher the CR content, the higher the *M_w_*. Additionally, the polar component of SFE is remarkably larger, and the adhesive work produced by the polar component is also greater and the adhesive property increased. This is mainly attributed to the addition of DA, which results in the radical disintegrating reaction of CR and the violent reaction between it and the base binder.

(4) For DHVRA, with the extension of mixing time, the storage stability increased significantly, and the softening point difference after 6 h is even less than 2 °C. According to the rheological test results, the high-temperature deformation resistance and temperature sensitivity are greatly reduced. With increasing CR content, the temperature sensitivity is improved, and it is still not as good as CRA, but much better than that of VA. Although the high-temperature performance is insufficient, the low-temperature relaxation ability of DHVRA was better than CRA and VA, and the DHVRA with CR content of 32% (32-DRA) has the best low-temperature relaxation ability. Therefore, 32-DRA has great advantages in the areas where the high requirements for low-temperature performance of pavement are needed.

## Figures and Tables

**Table 1:** Four components of different base asphalt.

Asphalt Type	Asphaltene (As)/%	Resins (R)/%	Saturates (S)/%	Aromatics (Ar)/%
SK-70	9.36	42.23	11.88	36.53
SK-90	10.28	31.80	8.50	49.42
Zhonghai-70	0.10	51.44	20.35	28.11
Zhoanghai-90	0.15	47.75	19.85	32.25

**Table 2:** Main indicators of SK-90.

Main Indicators		Test Results	Requirements
Penetration at 25 °C (100 g, 5 s)/0.1 mm		84.6	80~100
Ductility at 15 °C/cm		142.0	≥100
Softening Point/°C		48.7	≥44
RTFOT Residues	Mass Loss/%	0.5	≤0.8
	Residual penetration ratio/%	72.5	≥57
	Residual ductility/cm	22.6	≥8

**Table 3:** Main indicators of crumb rubber.

Main Indicators	Test Results	Requirements
Rubber Hydrocarbon/%	57.00	≥42
Carbon Black/%	30.27	≥28
Acetone Extractives/%	7.77	≤22
Ash Content/%	4.83	≤8
Fiber Content/%	0.01	<1
Metal Content/%	0.02	<0.03
Moisture Content/%	0.1	<1
Relative Density	1.257	1.10–1.30

**Table 4:** The parameters of long-term disintegrating tests.

Preparation Factors	Parameters
CR Content/%	24, 28, 32
DA Dosage/‰	7.5
Mixing Time/h	Samples were taken every 0.5 h in the first 3 h, and then 3 h, 4 h, 5 h, 8 h, 12 h and 18 h after 3 h
Heating Temperature/°C	190

**Table 5:** Long-term disintegrating stage division based on PGR.

Disintegrating Stage	CR Content/%	Mixing Time t/h	PGR/%
Stage I	32	≤1.0	>50
Stage II	24 28	1.0–2.5 1.0–2.0	10–30
Stage III	24 28 32	≥2.5 ≥2.0 ≥1.0	<5

**Table 6:** Long-term disintegrating phase division based on VDR.

Disintegrating Stage	Mixing Time t/h	VDR/%
Stage I	t ≤ 3	20–30
Stage II	3 < t ≤ 5	About 10
Stage III	>5	<10

**Table 7:** The differences in softening point between upper and lower asphalt binder materials.

Storage Time/h	Difference of Softening Point/°C		
	CRA	DHVRA (Mixed for 2 h)	DHVRA(Mixed for 3 h)
2	6.8	3.1	1.0
4	9.1	4.5	1.8
6	11.3	5.7	2.0

**Table 8:** R^2^ of penetration, RV and CR particle size of DHVRA.

Particle Size/µm	CR Content/%	R^2^ of Penetration	R^2^ of RV
Max Diameter	24	0.4141	0.8976
	28	0.3958	0.9678
	32	0.7829	0.9387
Mean Diameter	24	0.1080	0.7450
	28	0.2705	0.9017
	32	0.8090	0.9655

**Table 9:** Simplified table of different asphalt names.

Asphalt Types	Simplified Names
Virgin Asphalt	VA
Rubber asphalt with 24% CR stirred for 3 h without DA	24-RA
DHVRA with 24% CR and mixing for 3 h	24-DRA
DHVRA with 28% CR and mixing for 3 h	28-DRA
DHVRA with 32% CR and mixing for 3 h	32-DRA
Aged base asphalt (the names of other aged asphalts were simplified in the same way)	VA-R
DHVRA with 24% CR and mixing for 0.5 h (the names of other aged asphalts were simplified in the same way)	2405

**Table 10:** Contact angle test results of different asphalts with distilled water and glycol.

Asphalt Types	Contact Angle/°	
	Distilled Water	Glycol
VA	100.3	71
24-DRA	88.2	53.1
28-DRA	74.8	47.2
32-DRA	61.7	40.5
3215	69.3	45.4

**Table 11:** SFE parameters of different asphalt.

Asphalt Types	SFE Parameters/(mJ·m^−2^)		
	$\gamma_{s}^{d}$	$\gamma_{s}^{p}$	$\gamma_{s}$
VA	28.94	0.47	29.41
24-DRA	37.86	1.57	39.43
28-DRA	24.02	10.49	34.51
32-DRA	14.73	25.11	39.84
3215	18.66	16.65	35.31
24-RA	34.75	0.18	34.93

**Table 12:** Linear fitting equation and evaluation indexes of different asphalt binders.

Asphalt Types	Fitting Function: Y = ln(G*/sin*δ*) = −kln*T* + b	R^2^	Index k of Temperature Sensitivity	Failure Temperature/°C
2410	Y = −5.4506 lnT + 23.8807	0.998	5.4506	79.9
24-DRA	Y = −5.7799 lnT + 24.6491	0.999	5.7799	71.1
2440	Y = −5.7362 lnT + 24.3316	0.999	5.7362	69.5
2450	Y = −5.8152 lnT + 24.6314	0.999	5.8152	69.1
2810	Y = −4.6942 lnT + 20.7649	0.999	4.6942	83.4
28-DRA	Y = −5.1052 lnT + 22.0905	0.999	5.1052	75.7
2840	Y = −5.2486 lnT + 22.6003	0.998	5.2486	74.1
2850	Y = −5.3127 lnT + 22.7646	0.998	5.3127	72.6
3210	Y = −4.6008 lnT + 20.1498	0.999	4.6008	79.8
32-DRA	Y = −4.9600 lnT + 21.2567	0.999	4.9600	72.6
3240	Y = −4.9466 lnT + 21.1467	0.999	4.9466	71.9
3250	Y = −4.9804 lnT + 21.2751	0.999	4.9804	71.6
VA	Y = −7.5345 lnT + 31.4483	0.999	7.5345	65.0
24-RA	Y = −4.8802 lnT + 28.8243	0.998	4.8802	>180

## Data Availability

Not applicable.
